# Biochemical markers for the assessment of aquatic environment contamination

**DOI:** 10.2478/v10102-010-0034-y

**Published:** 2010-11

**Authors:** Marcela Havelková, Tomáš Randák, Jana Blahová, Iveta Slatinská, Zdeňka Svobodová

**Affiliations:** 1 University of Veterinary and Pharmaceutical Sciences Brno, Faculty of Veterinary Hygiene and Ecology, Brno, CZECH REPUBLIC; 2 University of South Bohemia České Budéjovice, Research Institute of Fish Culture and Hydrobiology Vodňany, Vodňany, CZECH REPUBLIC

**Keywords:** cytochrome P450, EROD, GST, GSH, river pollution

## Abstract

The need for assessment of aquatic ecosystem contamination and of its impact on water dwelling organisms was developed in response to rising aquatic environmental pollution. In this field study, liver enzymes of phase I and phase II of xenobiotic transformation, namely cytochrome P450, ethoxyresorufin-*O*-deethylase, glutathione-S-transferase and tripeptide glutathione were used to assess the contamination of the aquatic environment at different rivers in the Czech Republic. The indicator species selected was the male chub (*Leuciscus cephalus* L.) and male brown trout (*Salmo trutta fario*). Chemical analyses included also the assessment of the most important inductors of previously mentioned biochemical markers. The major inductors of monitored biomarkers are industrial contaminants which belong to a large group of organic pollutants (PCB, PAH, PCDD/F, DDT, HCH, HCB and OCS), persistent in the environment. Four different groups of river basins were assessed: the River Tichá Orlice and its tributary the Kralický brook; important tributaries of the River Elbe (the rivers Orlice, Chrudimka, Cidlina, Jizera, Vltava, Ohře and Bílina); major rivers in the Czech Republic (the rivers Lužnice, Otava, Sázava, Berounka, Vltava, Labe, Ohře, Svratka, Dyje, Morava and Odra) and the River Vltava. The use of the biochemical markers together with chemical analyses seems to be an effective way to monitor the quality of aquatic environment.

## Introduction

The aquatic environment has become an easily accessible disposal site for xenobiotics and pollutants such as organochlorine compounds. Contamination of water with industrial and agricultural pollutants influences the biochemical processes of aquatic organisms. An effective monitoring system using biochemical markers has been established to demonstrate these xenobiotics in the environment.

A biomarker often used in the studies assessing aquatic environment pollution is cytochrome P450 (CYP 450) system which has proved to be a very suitable method for biochemical and environmental monitoring of aquatic environment quality (Vindimian *et al*., 1991; Flammarion *et al*., 2002; Mayon *et al*., 2006; Koehler *et al*., 2007). Over 200,000 chemicals are metabolized by the CYP 450 family of enzymes which catalyses many different types of reactions including oxidations, reductions, and dehalogenations (Lewis, 2001). In fish, the class of CYP 450 isozymes responsible for the biotransformation of a myriad of xenobiotic compounds is the CYP1A subfamily (van der Oost *et al*., 2003) comprising two isoenzymes, CYP1A1 and CYP1A2 (Goksoyr and Forlin, 1992; Stegeman and Hahn, 1994). Regulation of the CYP1A family is mediated by the Ah (aryl hydrocarbon) receptor, which has a high affinity for the environmental pollutants, e.g. TCDD (2,3,7,8-tetrachlorodibenzo-*p*-dioxin), or planar PAH (polycyclic aromatic hydrocarbons) and PCB (polychlorinated biphenyls). Generally, the toxicity of a pollutant is related to the degree of its affinity to Ah receptor. On the other hand, chronical exposure or high levels of these pollutants in the environment can cause lower exposure response in synthesis of new CYP 450 in organisms living the contaminated environment (Brammel *et al*., 2004).

In addition to total CYP 450 assessment, a common method to examine the responses of the CYP1A isoenzymes is to determine its catalytic activity. The catalytic activity of an enzyme called ethoxyresorufin-*O*-deethylase (EROD) seems to be the most sensitive way used for the assessment of organic pollutants′ impact on fish health status (Goksoyr and Forlin, 1992; Whyte *et al*., 2000; van der Oost *et al*., 2003). The EROD activity which is closely connected to CYP1A1 is measured by following the increase in fluorescence of the reaction product resorufin (Burke and Mayer, 1974).

Another important liver biomarkers used for environmental pollution determination is enzyme glutathione S-transferase (GST) and tripeptide glutathione in its reduces form (GSH).

Intracellular tripeptide gluthatione presents the main molecule involved in the defense of cells against oxidative stress (Otto and Moon, 1996; Mannervik and Danielson, 1988). Catalyzed by the enzyme GST, conjugative reaction with GSH is the principal detoxification pathway for electrophilic xenobiotics. During the conjugative reaction, a thioether bond is formed between the cysteine remains of glutathione and the electrophilic substance, and the result of this process is usually a less reactive and more readily soluble product (Eaton and Bammler, 1999).

Both GST and GSH can also be used as biomarkers in fish, but their utility is less sustainable than in the case of CYP 1A (Van der Oost *et al*., 2003). Spectrum of potential GST inducers is variable, increased overall GST activity has been demonstrated in the liver of various fish species after the exposure to PCBs (Perez-Lopez *et al*., 2000; Perez-Lopez *et al*., 2002; Schmidt *et al*., 2004), PAHs (Noble *et al*., 1998; Henson *et al*., 2001), and some pesticides (Frasco and Guilhermino, 2002).

The aim of the presented study was to use selected liver biochemical markers (CYP 450, EROD, GST, GSH) measured in two indicator fish species – brown trout (*Salmo trutta fario*) and chub (*Leuciscus cephalus* L*.)* to assess contamination levels at selected rivers in the Czech Republic. The results of these biochemical analyses were compared with the results of specific inductors of measured biomarkers – organic pollutants in fish muscle and sediment samples. Contamination levels of the selected locations were assessed on the basis of the results, and the localities most polluted and endangering health of water organisms were revealed.

## Materials and Methods

This study was conducted in accordance with national and institutional guidelines for the protection of human subjects and animal welfare (Animal protection law No. 246/92 Collection of Law).

This study is a review of four previous studies (Havelkova *et al*., 2007; Havelkova *et al*. 2008a; Havelkova *et al*., 2008b; Slatinska *et al*., 2008).

Brown trout (*Salmo trutta fario*) and chub (*Leuciscus cephalus* L.) were chosen as indicator fish species. Brown trout was chosen for the River Tichá Orlice and its tributary Králický brook because fish reproductive problems and spawning disturbances were detected in this fish species from this locality before. Chub was used as the indicator fish at all the others localities (that means the River Elbe and its tributaries, main rivers in the Czech Republic and the River Vltava) because chub represents an omnivorous species and was available at all sampling, both polluted and non polluted, sites.

### Collection of tissue samples

#### 1. The river Tichá Orlice

**Figure 1:**
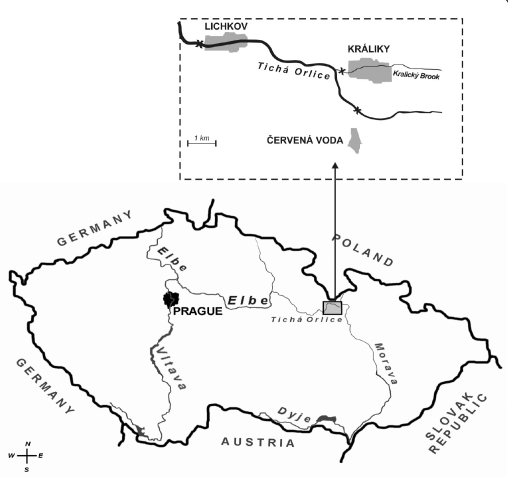
Map of the Czech Republic and location of sampling area.

Fish were examined at three sites along the River Tichá Orlice (Červená Voda, Králíky and Lichkov). The main characteristics of fish captured in individual locations are summarized in Table 1. The sites Červená Voda, Králíky and Lichkov, are 103, 100 and 93 km east of the junction of the Tichá Orlice with the Elbe (Figure 1). Individual sites were separated by cross barriers. Červená Voda was chosen as a control site because it showed minimum loads in previous study (Kolarova *et al*. 2005). Samples of muscle and liver from male brown trout (*Salmo trutta fario*) were obtained in June 2003. Bottom sediment samples were collected at the same time from each location. Separate liver samples were collected and processed for each fish, while individual muscle samples were pooled on site to create a combined sample for chemical analysis.

**Table 1:** The main characteristics of sampled brown trout males (*Salmo trutta fario*) population.

Locality	Weight (g) (mean ± SD)	Mean age (years) (min–max)
Červená Voda	91±12	2.1 (2–3)
Králíky	118±66	2.4 (2–4)
Lichkov	119±43	2.5 (2–4)

#### 2. The main tributaries of the river Elbe

**Figure 2:**
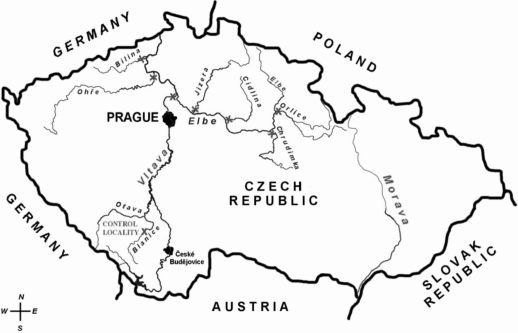
Map of the Czech Republic and location of sampling sites and the control site.

In summer (from May to June) 2006, male chub were caught in the Rivers: the Orlice, the Chrudimka, the Cidlina, the Jizera, the Vltava, the Ohře, and the Bílina, and at a control location on the River Blanice. The individual locations are shown in Figure 2. At each location, 3–10 chub were caught. The biometric characteristics of these fish are given in Table 2. Individual liver samples were taken for analysis of biochemical markers (CYP P450, EROD) and individual muscle samples for organic pollutants (polychlorinated biphenyls, hexachlorbenzene, hexachlorcyclohexan, octachlorstyrene and DDT and its metabolites) concentrations.

**Table 2:** Biometric characteristics of male chub (*Leuciscus cephalus* L.).

Location	(n)	Weight (g) mean±SD	Age (years) (mean of the total) (min–max)
Orlice	6	323±39	3.8 (3–4)
Chrudimka	10	180±11	3.2 (3–4)
Cidlina	9	238±53	3.7 (2–5)
Jizera	3	377±189	4.0 (3–5)
Vltava	7	290±30	3.3 (3–4)
Ohře	10	541±64	4.8 (3–7)
Bílina	10	121±18	2.3 (2–3)
Blanice (Control Location)	10	339±39	4.8 (3–6)

n = number of fish examined

#### 3. The major rivers in the Czech Republic

**Figure 3:**
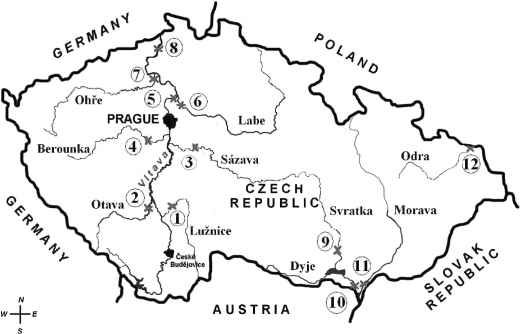
Map of the Czech Republic and locations of sampling sites.(1. Lužnice (Bechyně), 2. Otava (Topělec), 3. Sázava (Nespeky), 4. Berounka (Srbsko), 5. Vltava (Zelčín), 6. Labe (Obříství), 7. Ohře (Terezín), 8. Labe (Děčín), 9. Svratka (Židlochovice), 10. Dyje (Pohansko), 11. Morava (Lanžhot), 12. Odra (Bohumín)).

In May and June 2006, male chub were caught at, or near, the mouths of 11 major rivers in the Czech Republic: The Lužnice (Bechyně), Otava (Topělec), Sázava (Nespeky), Berounka (Srbsko), Vltava (Zelčín), Labe (Obříství), Ohře (Terezín), Labe (Děčín), Svratka (Židlochovice), Dyje (Pohansko), Morava (Lanžhot), Odra (Bohumín). The municipality near which the samples were taken is given in brackets. The sampling sites are shown in Figure 3. At most of the sites, 8 male chub were captured by electrofishing. The number and biometric characteristics of fish captured are given in Table 3. Individual liver samples were taken for analysis of biochemical markers (CYP 450, EROD, GST, GSH). Muscle samples were pooled on site to create a combined sample for chemical analyses of polychlorinated dibenzo-*p*-dioxins (PCDD), polychlorinated dibenzofurans (PCDF) and polychlorinated biphenyls (PCB).

**Table 3:** Characteristics of male chub (*Leuciscus cephalus* L.) from sampling sites.

Location River (Municipality) (dist. from site to mouth of river)	n	Weight±SD (g)	Age (years) mean (range)
Lužnice (Bechyně) (11 km)	8	614.4±155.0	4.9 (4–6)
Otava (Topělec) (20 km)	9	360.6±253.0	4.2 (3–6)
Sázava (Nespeky) (27.5 km)	8	278.1±63.9	3.8 (3–4)
Berounka (Srbsko) (29 km)	8	292.5±99.5	3.6 (3–5)
Vltava (Zelčín) (5 km)	10	383.5±199.3	4.0 (3–7)
Labe (Obříství) (122 km)*	8	306.3±144.9	4.1 (3–6)
Ohře (Terezín) (3 km)	10	540.5±201.8	4.3 (3–7)
Labe (Děčín) (21 km)*	8	546.3±211.0	4.8 (4–7)
Svratka (Židlochovice) (23 km)	8	243.8±94.2	3.5 (3–4)
Dyje (Pohansko) (16 km)	3	626.7±558.2	4.7 (3–7)
Morava (Lanžhot) (9.5 km)	8	304.4±110.6	3.1 (2–4)
Odra (Bohumín) (9 km)**	8	149.4±78.1	2.8 (2–4)

* Distances between Labe sites and the border with Germany

** Distance between Odra site and the border with Poland

n = number of fish

#### 4. The river Vltava

**Figure 4:**
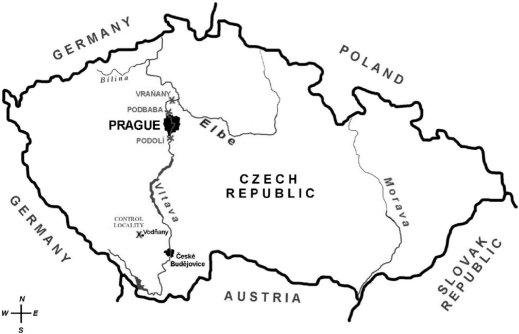
Sample-collecting locations along the River Vltava and the control locality.

Between ten and twelve male chub were captured at each location from April to May 2005 by electrofishing. Biometric details of fish are summarized in Table 4. The chub were caught at three locations along the River Vltava and at the control site (a pond at Vodňany). The locations along the River Vltava were Podolí (upstream of Prague), Podbaba (downstream of the Prague, downstream of the waste water treatment plant) and Vraňany (downstream of Kralupy nad Vltavou, a chemical factory). Fish from the pond at Vodňany were used as a control group. The sampling sites are shown in Figure 4.

**Table 4:** Biometric details of chub (*Leuciscus cephalus* L.) used in the study.

Location	n	Mean weight ±S.D (g)	Age (min–max) (years)
Vodňany pond (control locality)	12	279±144	3–6
Podolí (upstream of Prague)	10	331±96	3–5
Podbaba (downstream of Prague)	10	290±146	3–8
Vraňany (downstream of Prague)	12	718±296	4–8

All fish were captured by electrofishing and killed by severing the spinal cord after stunning. They were weighed and measured, and their scales were collected for age determination. Immediately after death, individual liver samples of about 2 g of weight were collected from each fish for the determination of biochemical markers. Liver samples were immediately frozen in liquid nitrogen and stored at –80°C until enzyme assays were performed. Samples of fish muscle were collected for chemical analyses of specific inductors of biomarkers. Muscle samples were placed in thermo-boxes filled with dry ice and later frozen and stored at –18°C until analysis. The PAH levels in bottom sediments at all investigated locations were determined in samples of fine inconsistent sediments withdrawn close to the river bank at a depth of 0.05–0.15 m. Metal core samplers were used to collect samples. Sediments samples were homogenized and transported refrigerated (4°C) to the laboratory, where they were stored at –18°C until analysis (Siroka *et al*., 2005). Collection of bottom sediment samples was carried out according to the Czech norm ČSN ISO 5667-12.

### Determination of cytochrome P450 and EROD activity in liver samples

Before the enzymes were assayed, microsomal protein concentrations were determined in each sample according to Lowry (1951). Depending on protein concentrations, the required amounts of microsomal suspension were taken for the quantification of CYP 450 content and the determination of EROD activity.

Before the analysis, a sample of liver tissue was placed into a homogenization buffer (0.25 M saccharose, 0.01 M TRIS and 0.1 mM EDTA, pH 7.4) and homogenized. Homogenized samples were two times centrifuged at 10,000 g for 20 min at a temperature 4°C and 100,000 g for 60 min at 4°C, respectively. The suspension was put into eppendorf tubes and stored at –80°C until the enzymatic analyses.

Visible light spectrophotometry (390–490 nm) was used to determine the total quantity of CYP 450. Measurements were made after cytochrome reduction by sodium dithionite and after the complex with carbon monoxide was established. The method is described in detail in Siroka *et al*. (2005). Cytochrome P450 values were homogenous in more than 90% of measured samples (coefficient of variation less than 10%). Each sample was analyzed in triplicate and the mean value was used for statistical analysis.

The EROD catalytic activity was determined by fluorescence spectrophotometry, detection limit: 2 pmol/mg/min protein. The spectrophotometer monochromators are set at 535 nm and 585 nm for excitation and emission wavelenght, respectively. In the presence of NADPH (nicotinamide adenine dinucleotide phosphate), EROD enzymatic activity converts the substrate ethoxyresorufin to resorufin, which is a fluorescent product. This method is in detail described in Siroka *et al*. (2005). Measurements were made using the Perkin-Elmer Fluorescence Spectrophotometer 203. EROD values were homogenous in more than 90% of measured samples (coefficient of variation less than 15%). Each sample was analyzed in triplicate and the mean value was used for statistical analysis.

### Determination of GSH and GST in liver samples

Before analysis, frozen liver samples were extracted with a phosphate buffer. Phosphate buffer (pH 7.2) was added to samples, which were homogenized and then centrifuged for 10 min at 2,400 g and 4°C. The supernatant was used for determination of glutathione-S-transferase (GST), glutathione (GSH) and protein concentration. The catalytic activity of GST was measured spectrophotometrically by a modified method of Habig *et al*. (1974) using the biochemical analyzer Cobas Emira (at 340 nm). The specific activity was expressed as nmol of formed product per minute per milligram of protein.

Tripeptide glutathione was determined by the method of Ellman (1959) using the biochemical analyzer Cobas Emira. Trichloroacetic acid was added to supernatant to precipitate protein. This was vortexed for 5 min and, after 15 min of incubation at the room temperature, centrifuged for 10 min at 2,400 g and 4°C. The supernatant and reaction agents (buffer – 0.8 M Tris/HCl, 0.02 M EDTA pH 7.2 and 0.01 M 2,2-dinitro-5,5-dithiobenzoic acid in ethanol) were pipetted into sample cells. Absorbance of coloured product was determined at 414 nm and concentrations (nmol/mg protein) calculated according to a standard calibration.

The protein concentration was determined by the Bicinchoninic Acid Protein Essay Kit (Sigma-Aldrich) using bovine serum albumin as standard (Smith *et al*., 1985). Each sample was analyzed in triplicate (for both GST and GSH) and the mean value was used for statistical analysis.

### Determination of organic pollutants in muscle samples

Polychlorinated biphenyl (PCB) congeners – IUPAC numbers 28, 52, 101, 118, 138, 153, 180, hexachlorocyclohexane (HCH), hexachlorbenzene (HCB), octachlorostyrene (OCS) and DDT and its metabolites (DDE and DDD) were determined in fish muscle samples by two-dimensional capillary gas chromatography employing two parallel columns of equal dimensions, differing in selectivity, (DB-5 and DB-17) and two electron-capture detectors. Isolation of target analytes from fish muscle was carried out by Soxhlet extraction into hexane:dichloromethane (1:1, v/v) solvent mixture. The purification of extracts was performed by Gel Permeation Chromatography on a Bio-Beads S-X3 column and mobile phase ethylacetate:cyclohexane (1:1, v/v) (Hajslova *et al*., 1995).

### Determination of PCDD/PCDF in muscle samples

The pooled samples of fish muscle were homogenized and dried by lyophilization. Internal standards (15 13C12, labeled PCDD/PCDF) were added to the lyophilized sample. The samples were Soxhlet-extracted with a 3:1 hexane:acetone mixture for 24 h. Fat removal, consequential clean up, and fractionation were performed by a combination of dialysis with a semipermeable membrane and a modified EPA 1613 method (Grabic *et al*., 2000).

PCDD/PCDFs were analyzed using gas chromatography/high-resolution mass spectrometry (Thermo Electron MAT95XP) in MID scan mode (Grabic *et al*., 2000).

Toxic equivalents (TEQs) for fish were used to express levels of PCDD/PDCF in fish muscle, as reported by Van den Berg *et al*. (1998).

The laboratory is accredited by Czech Accreditation Institute in accordance to ISO 17 025 norm. The method was validated using certified reference materials NIST 1588a, NIST 1944, WMF, WMS (Wellington Laboratories). In accordance with our standard operating procedures, we obtain recoveries of IS ranging from 50–130%.

### Determination of PAH in sediments

Before the assay, sediments obtained from one location were mixed and a composite sample was prepared. Polycyclic aromatic hydrocarbons were determined using reversible high performance liquid chromatography and fluorescence detection. The following 15 PAHs were found in bottom sediments: fluorene, naphthalene, acenaphthene, fenanthrene, anthracene, fluoranthene, chrysene, pyrene, benzo(a)anthracene, benzo(b)fluoranthene, benzo(k)fluoranthene, benzo(a)pyrene, indeno(1,2,3-c,d)pyrene, benzo(g,h,i)perylene and dibenzo(a,h)anthracene. These 15 PAHs are European Union priority congeners (EPA 610). The sediment sample was determined according to Hosnedl *et al*. (2003). Recovery of PAHs from sediments exceeded 80% for all compounds at concentration level of individual PAHs 100 ng/g d.w. The laboratory is accredited by Czech Accreditation Institute in accordance with ISO 17025 norm. In accordance with our standard operating procedures, we obtain recoveries of IS ranging from 50–125%.

### Statistical analysis

Statistical analysis of the data was performed using the program STATISTICA 6.1 for Windows (StatSoft CR). Because normality of the distribution of the data obtained was not demonstrated, nonparametric techniques were used. The Kruskal-Wallis test was used to compare contaminant concentrations as well as biochemical markers of contamination found at individual locations. If the Kruskal-Wallis test showed significant differences between locations (p<0.05), multiple comparisons of all location pairs were subsequently performed. The Spearman's rank correlation coefficient was used to monitor the relationship between selected biomarkers and exogenous substances both within locations and among them.

## Results

### 1. The River Tichá Orlice

#### Results of biochemical markers determination

The lowest values of CYP 450 content and EROD activity in brown trout liver were found at the site Červená Voda, while the highest values of these markers were determined at Králíky.

The highest levels of CYP 450 (0.114 ± 0.091 nM/mg protein, mean ± SD) were found at Králíky, lower levels were found at Lichkov (0.085 ± 0.061 nM/mg protein), and the lowest CYP 450 levels were at the control site Červená Voda (0.077 ± 0.041 nM/mg protein). However these differences in CYP 450 values between sites were not statistically significant.

On the other hand, the differences in EROD values between sites were significant (p<0.001). The highest EROD activity in fish liver was found at Králíky (861.9 ± 561.04 pmol/min/mg protein, mean ± SD), lower EROD activity was found at Lichkov (359.0 ± 107.91 pmol/min/mg protein), and the lowest EROD activity was found at the control site Červená Voda (196.8 ± 148.21 pmol/min/mg protein). Multiple comparison showed a significant difference in EROD values between Králíky and Červená Voda (p<0.001). A positive significant correlation between EROD activity and CYP 450 content was found at Červená Voda (r_s_=0.787, p=0.007). This relationship was not significant at either Králíky or Lichkov.

#### Results of chemical analyses

The pollutants found in muscle of brown trout caught at the monitoring sites included seven PCB congeners, HCH (as a sum of three congeners), HCB, DDT including its metabolites and OCS, and their values are presented in Table 5. The presence of PAH (as a sum of 15 selected PAHs) in the sediment is also demonstrated. No correlation between PCB and biochemical markers from individual sites was observed.

**Table 5:** Content of persistent organic pollutants in chub (*Leuciscus cephalus* L.) male population (μg/kg of muscle, w.w.).

Locality	PCB^a)^	HCH^b)^	HCB	OCS	DDT^c)^	PAH^d)^
Červená Voda	10 ± 0.2	0.14 ± 0.04	0.70 ± 0.06	0.04 ± 0.01	40 ± 2.2	2800
Králíky	48 ± 3.6	0.30 ± 0.01	2.6 ± 0.14	0.05 ± 0.01	50 ± 20.6	16360
Lichkov	27 ± 6.1	0.20 ± 1.04	1.20 ± 0.03	0.05 ± 0.01	27 ± 5.1	1380

a) sum of 7 indicator congeners (28, 52, 101, 118, 138, 153, 180);

b) sum of HCH isomers (α, β, γ);

c) sum of DDT and its metabolites (*o,p′*- DDE; *p,p*′- DDE; *o,p′*- DDD; *p,p′*- DDD; *o,p′*-DDT; *p,p′*- DDT);

d) (ng/g dry matter)

### 2. The main tributaries of the river Elbe

#### Results of biochemical markers determination

The highest total CYP 450 in fish liver was detected in the River Vltava (mean 0.241 ± 0.148 nmol/mg protein), while the lowest concentrations were found in the River Blanice (mean 0.152 ± 0.091 nmol/mg protein) and the River Orlice (mean 0.120 ± 0.089 nmol/mg protein). Statistical analysis showed no significant differences in CYP 450 concentrations in the liver of indicator fish between locations.

**Figure 5:**
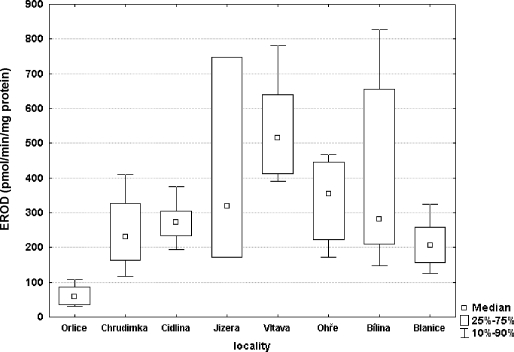
EROD activity in liver samples of male chub.

The highest EROD activity in fish liver was assessed in the River Vltava (mean 576.4 pmol/min/mg protein) and the lowest level in the River Orlice (mean 63.05 pmol/min/mg protein) (Figure 5). Samples from the River Blanice, which was established as the control site, had surprisingly mean value of 213.7 pmol/min/mg protein. Statistical analysis of EROD activity showed a significant difference (p<0.05) between the Blanice control and the River Vltava, and also a significant difference between the River Orlice and the River Vltava (p<0.01), the River Bílina (p<0.01) and the River Ohře (p<0.05).

Significant correlations (R=0.687) between EROD activity and CYP 450 were found in fish from all locations at p<0.01.

#### Results of chemical analyses

Results of chemical monitoring are summarized in Table 6.

**Table 6:** Content of persistent organic pollutants in male chub (Leuciscus cephalus L.) (μg kg-1 of muscle, w.w.).

LOCATION	PCB^a)^	DDT^b)^	HCB	HCH^c)^	OCS
Orlice	15.1±2.66	10.6±1.54	1.51±0.23	0.31±0.1	0.028±0.004
Chrudimka	9.1±1.18	13.0±1.27	1.57±0.10	0.22±0.04	0.041±0.003
Cidlina	13.7±3.24	8.1±1.67	0.52±0.05	0.45±0.10	0.072±0.02
Jizera	51.9±23.73	23.2±10.21	1.22±0.36	0.20±0.02	0.24±0.13
Vltava	46.1±7.11	25.1±3.23	1.55±0.19	0.21±0.03	0.12±0.02
Ohře	68.6±10.95	44.0±5.51	2.82±0.38	0.56±0.21	0.46±0.23
Bílina	31.9±2.92	14.4±1.73	2.93±0.38	0.32±0.01	0.10±0.02
Blanice (Control location)	10.8±1.06	35.0±4.09	1.75±0.20	0.18±0.03	0.03±0.008

a) sum of 7 indicator congeners (28, 52, 101, 118, 138, 153, 180);

b) sum of DDT (*o,p*′- DDE; *p,p*′- DDE; *o,p*′- DDD; *p,p*′- DDD; *o,p*′-DDT; *p,p*′- DDT);

c) sum of HCH isomers (α, β, γ)

The highest concentrations of pollutants monitored were revealed in fish from the River Ohře and the River Vltava. The lowest concentrations were found in the River Blanice and in the River Chrudimka.

PCB concentrations in muscle of indicator fish from the River Ohře (p<0.01) and the River Vltava (p<0.05) were significantly higher than those found in the fish from the River Blanice. Significant differences were also found between the River Chrudimka and the River Vltava (p<0.01) and the River Ohře (p<0.05), and between the River Ohře and the River Cidlina (p<0.05).

### 3. The major rivers in the Czech Republic

#### Results of biochemical markers determination

**Figure 6:**
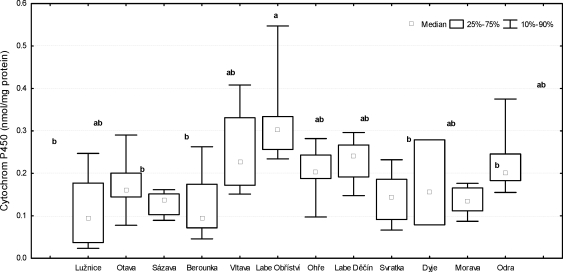
Content of cytochrome P450 in male chub (*Leuciscus cephalus* L.) livers. Significant differences (p<0.05) are indicated by alphabetic superscript (a, b).

The highest CYP 450 levels in liver were found in fish from the River Labe (Obříství) (0.32 ± 0.10 nmol/mg protein) while the lowest concentration in the River Lužnice (0.11 ± 0.08 nmol/mg protein) (Figure 6). Statistical analysis of CYP 450 levels showed significant differences between the location Labe (Obříství) and following rivers: the Lužnice, the Sázava, the Berounka, the Svratka and the Morava (p<0.05).

**Figure 7:**
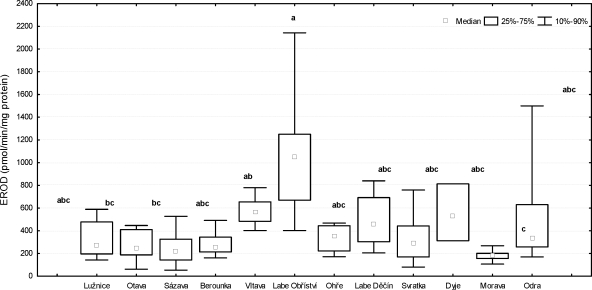
EROD activity in male chub (*Leuciscus cephalus* L.) livers. Significant differences (p<0.05) are indicated by alphabetic superscript (a, b, c).

The highest EROD activity in liver was detected in fish from the location Labe (Obříství) (1061).38 ± 545.51 pmol/min/mg protein) and the lowest EROD activity in the River Morava (183.04 ± 48.20 pmol/min/mg protein) (Figure 7). Significant differences were found (p<0.05) between the Labe (Obříství) and the River Otava, the River Sázava, and the River Morava. A significant difference (p<0.05) was also found between the River Morava and the River Vltava.

**Figure 8:**
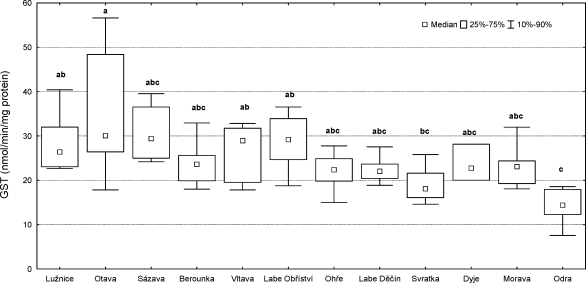
GST catalytic activity in male chub (*Leuciscus cephalus* L.) livers. Significant differences (p<0.05) are indicated by alphabetic superscript (a, b, c).

The highest GST catalytic activity in fish liver was detected in fish from the Otava (35.39 ± 13.35 nmol/min/mg protein). The lowest was in those from the Odra site (14.43 ± 3.80 nmol/min/mg protein). Statistical analysis of GST activity showed significant differences between the location Odra and the locations Lužnice, Otava, Vltava, and Labe (Obříství) (p<0.05), and also between the Svratka and the Otava (p<0.05) (Figure 8).

**Figure 9:**
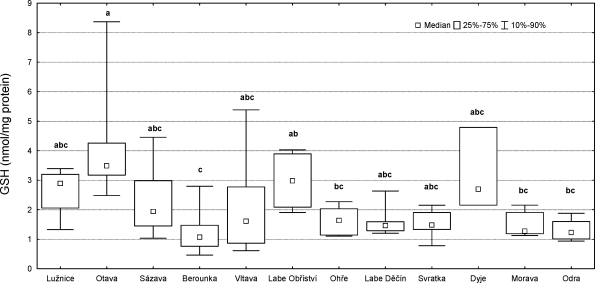
Content of tripeptide GSH in male chub (*Leuciscus cephalus* L.) livers. Significant differences (p<0.05) are indicated by alphabetic superscript (a, b, c).

The highest GSH tripeptide content in fish liver was detected in the River Otava (4.29 ± 2.10 nmol/mg protein), the lowest GSH content was in the River Berounka (1.23 ± 0.73 nmol/mg protein). Statistical analysis of GSH content in fish liver showed significant differences between the River Otava and: the River Berounka, the River Ohře, the River Morava, and the River Odra (p<0.05); statistical analysis also showed significant differences between the River Labe (Obříství) and the River Berounka (p<0.05) (Figure 9).

Significant correlations (R=0.571) of EROD activity with CYP 450 and of GST with GSH (R=0.595) in liver tissue were found in fish from all locations (p<0.01).

#### Results of chemical analyses

**Figure 10:**
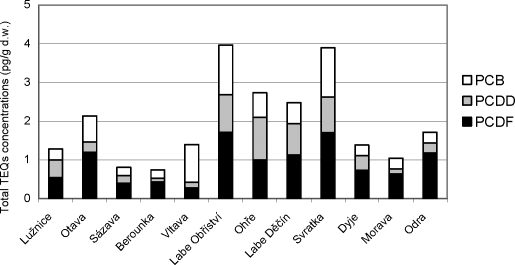
Total TEQ concentrations for PCDD/PCDFs and PCBs (pg/g d. w.) in male chub (*Leuciscus cephalus* L.) muscle.

The concentrations of PCCD/PCDF TEQs in male chub muscle ranged from 0.10 (Berounka) to 1.10 pg/g dry weight (d.w.) (Ohře) and from 0.27 (Vltava) to 1.71 pg/g d.w. (Labe Obříství and Svratka), respectively. The concentrations of PCB TEQs in male chub muscle ranged from 0.21 (Berounka) to 1.28 pg/g d.w. (Labe Obříství). Total TEQs concentrations (Σ of PCDD/PCDF and PCB TEQs concentrations) in male chub muscle ranged from 0.74 (Berounka) to 3.97 pg/g d.w. (Labe Obříství). The toxic equivalents of analysed chemicals are shown in Figure 10. No correlation with biochemical markers from individual sites was performed because of the composite chub muscle samples.

### 4. The river Vltava

#### Results of biochemical markers determination

Results of the assessment of the phase I enzymes (CYP 450, EROD) in fish liver from four locations showed no statistically significant differences among all investigated locations. The highest level of CYP 450 (median 0.18 nmol/mg protein) was found in Vraňany samples (under chemical plant downstream of the Prague conurbation) and the lowest level (median 0.15 nmol/mg protein) was found in Podolí (upstream the city). Increased EROD activity was found in chub liver from Podbaba (median 101.37 pmol/min/mg protein) compared to activity found in samples from Podolí (median 60.75 pmol/min/mg protein). But as well as CYP 450 levels, EROD activity levels found in fish liver from the four locations were not significantly different among sampling sites.

On the contrary, results of GST and peptide GSH seem to be more variable. The highest level of GST activity was found in chub liver from Podbaba (median 42.82 nmol/min/mg protein) while the lowest activity was found at control locality Vodňany (median 12.60 nmol/min/mg protein). Glutathione S-transferase activity in samples from Podbaba was significantly higher (p<0.01) than GST levels recorded at Vodňany, Podolí or Vraňany. The highest GSH concentration in chub liver was found in fish from Podbaba (median 8.01 nmol/mg proteins), and the lowest concentration was found at control locality Vodňany (median 0.97 nmol/min/mg protein). Glutathione concentrations in liver from Podbaba and Vraňany were significantly higher (p<0.001 and p<0.01, respectively) than those from Vodňany. Glutathione concentrations in liver from Podbaba were significantly higher (p<0.001) than GSH concentrations at Podolí.

**Figure 11:**
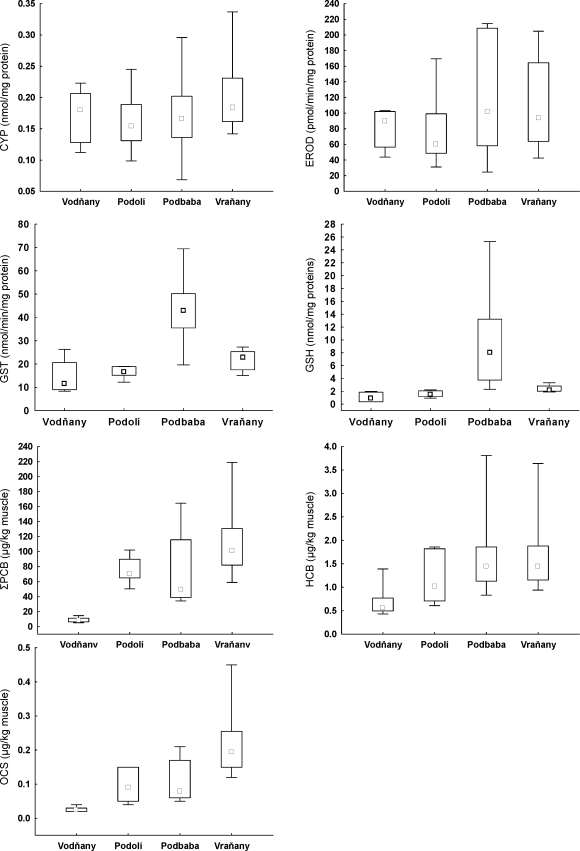
Biochemical characteristics and POPs concentrations in muscle samples of chub from three locations along the river Vltava and from control locality Vodňany

The main characteristics of all measured biochemical markers in chub liver are summarized in Figure 11.

#### Results of chemical analyses

Results of chemical monitoring are summarized in Table 7 and Figure 11. In all locations studied, concentrations of the selected chemical pollutants measured in chub muscle (PCB, HCB and OCS) were significantly higher (p<0.01) than those in samples from the control location, Vodňany.

**Table 7:** Content of 15 PAHs in bottom sediments.

PAH compounds*	Location			
	
	Vodňany	Podolí	Podbaba	Vraňany
fluorene	7.3	163.9	192.9	41.6
naphthalene	4.4	59.6	64.0	12.3
acenaphtene	5.0	117.6	120.4	8.1
fenanthrene,	221.9	62.7	1084.2	1460.5
anthracene,	7.9	284.9	317.4	24.1
fluoranthene	146.6	1992.9	2329.5	423.8
pyrene	114.7	1521.2	1792.7	234.8
benzo(a)anthracene	64.6	984.8	1062.2	117.6
chrysene	59.3	810.2	963.4	169.8
benzo(b)fluoranthene	74.3	965.1	905.4	362.4
benzo(k)fluoranthene	37.5	440.4	506.1	145.0
benzo(a)pyrene	68.6	1082.1	954.6	108.3
indeno(1,2,3-c,d)pyrene	71.4	767.5	795.7	137.5
benzo(g,h,i)perylene	59.6	483.1	643.8	122.2
dibenzo(a,h) anthracene	7.5	88.9	86.7	13.6
∑ PAH	791	10846	12195	2143

* PAHs are reported as μg kg-1 dry matter of bottom sediment

The location most contaminated by PCB was Vraňany where median of PCB concentration founded in chub muscle samples was 123.10 µg/kg muscle, wet weight (w. w.). The lowest PCB concentration was found at the control location (Vodňany) (median 8.15 µg/kg muscle, w. w.). The PCB concentrations in muscle samples of chub from Podolí, Podbaba, and Vraňany were significantly higher (p<0.001) than those from the control location (Vodňany).

The results of HCB determination in chub muscle obtained from the locations Podbaba and Vraňany gave the same high value (median 1.45 µg/kg muscle, w. w.), these two locations present the most contaminated sites. The lowest contamination was found at the control site Vodňany (median 0.56 µg/kg muscle, w. w.). The HCB concentrations in chub muscle from Podbaba and Vraňany were significantly higher (p<0.01) than those from Podolí and from the control site Vodňany.

Speaking about OCS, the situation is very similar to that in PCB – the levels of OCS in chub muscle samples were 0.20 µg/kg muscle w. w. (median value) at Vraňany and the lowest OCS level was again found at the control site (Vodňany) (median 0.03 µg/kg muscle). The OCS concentrations in chub muscle from Podolí, Podbaba, (p<0.05) and Vraňany (p<0.001) were significantly higher than those from the control location.

The concentrations of individual PAH congeners and the sum of all congeners in bottom sediments all measured locations are shown in Table 7. The highest values of ∑ 15 PAH were found at Podbaba (12,195 µg/kg dry matter of bottom sediment) and Podolí (10,846 µg/kg dry matter of bottom sediment). PAH concentrations in bottom sediments from Podolí, Podbaba, and Vraňany were significantly higher (p<0.001 and p<0.05, respectively) than those from the control location.

Spearman rank correlations between biochemical markers and chemical monitoring in indicator fish are summarized in Table 8.

**Table 8:** Spearman rank correlation between biochemical characteristics and POPs concentrations in muscles.

	CYP	EROD	GST	GSH	PCB (muscle)	HCB (muscle)	OCS (muscle)
**CYP**		**0.344**	0.079	0.089	0.086	0.028	0.085
**EROD**			0.191	0.253	0.168	0.129	0.209
**GST**				**0.718**	0.261	**0.478**	**0.433**
**GSH**					**0.390**	**0.411**	**0.481**
**PCB (muscle)**						**0.766**	**0.907**
**HCB (muscle)**							**0.813**
**OCS (muscle)**							

Statistically significant correlations at level ɑ=0.05 are given in bold.

## Discussion

Selected biochemical markers of phase I and phase II of xenobiotic metabolism were used to assess pollution in the rivers in the Czech Republic. These biomarkers are widely used in a number of studies which assess aquatic environmental pollution all around the world, e. g. Foster *et al*. (2001) – the Columbia River (USA); Al-Arabi *et al*. (2005) – the Karnaphuly River (Bangladesh); Siroka *et al*. (2005) – the River Elbe (the Czech Republic), Koehler *et al*. (2007) – the River Mures (Rumania); Parente *et al*. (2008) – the River Guandu (Brazil).

The relationship between levels of biochemical markers in fish liver and the ambient level of organic pollutants in fish muscle and bottom sediment were demonstrated in the presented study.

The major inducers of commonly monitored biomarkers are contaminants that belong to a large group of organic pollutants. They are PAH, PCB, and PCDD/PCDF and all of them are highly persistent in the environment. Despite efforts to reduce intentional and incidental releases, organic pollutants are frequently detected in environmental samples. Increased levels of organic pollutants result in an increase in values of corresponding biochemical markers. Monitored biochemical markers showed differences in contamination levels between individual sites, and the levels of organic pollutants in sediments confirmed these differences.

High levels of biochemical markers and their inductors were found on the River Tichá Orlice – the Králíky site, on the Rivers Elbe, Ohře and Bílina and on the River Vltava. The main sources of the organic pollutants on these rivers are industry and anthropogenic pollution, e. g. the source of pollution at Králíky site is a plant near Králíky which has manufactured electronics and which has been located on the Kralický brook – the tributary of the River Tichá Orlice (Svobodova *et al*. 2004; Havelkova *et al*., 2008b). Another case – the River Elbe – belongs to highly polluted European rivers (Heininger and Pelzer, 1998; Lehmann and Rode, 2001). Numerous chemical plants are located along its banks as well as along the tributaries of the upper Labe in the Czech Republic. The highest contamination was found at localities situated downstream from major chemical manufacturing plants (Labe Obříství, Labe Děčín); presumably as a result of dilution, the toxic substances are homogeneously distributed along the River Labe. The tributaries which contaminate the River Elbe the most were the rivers Ohře and Bílina. Anthropogenic pollution is another important factor influencing aquatic environment. The negative impact of the Prague conurbation on the River Vltava was determined in the previous study (Siroka *et al*., 2005; Randak *et al*, 2006; Slatinska *et al*., 2008) – the localities situated downstream of Prague were highly contaminated compared to the localities upstream of Prague.

However, some results of the presented study suggest that increased PCB and PAH concentrations do not always result in an increase in CYP 450 levels (the case of the River Vltava where concentrations of phase I enzymes are very similar at all tested locations). This is in agreement with other field studies, where the chub was used as an indicator organism (Flammarion *et al*., 2002; Krca *et al*., 2007). Results similar to that achieved in our study have been reported in chub by Siroka *et al*. (2005). In their study of contamination of the River Elbe and the River Vltava (the Czech Republic), they failed to find any significant increases in the activity of CYP450 or EROD even at demonstrably increased PCB and PAH concentrations. The contents both of measured biochemical markers and organic pollutants were comparable to our results.

On the other hand, there are known certain substances that can inhibit the inductive activity. The specific inhibitors in aquatic environment are metals (e.g. Cu, Zn, Pb, Cd, Cr or Ni), nonplanar PCB congeners or detergents which may act as specific CYP 450 inhibitors (Forlin *et al*. 1986; Boon *et al*. 1992; Stien *et al*. 1997; Bozcaarmutlu and Arinc 2004; Brammell *et al*. 2004; Henczova *et al*. 2006; Sen and Semiz, 2007). Also high levels of the inductors in the environment or chronic exposure to organic pollutants may reduce the organism′s response to the contamination, instead of inducing total CYP 450 (Stegeman *et al*. 1997; Schlezinger and Stegeman 2001). i.e. it may be the cause of an insufficient response regarding the synthesis of new CYP 450 (Brammel *et al*. 2004).

Measures of phase II enzymes may be useful in the context of the balance between phase I activation and phase II detoxification. Major inductors of the GST enzyme and GSH are also considered to be organic pollutants: PCBs, PAHs and PCCD/PCDFs (Gadagbui and Goksoyr, 1996; Otto and Moon, 1996). A positive correlation between GST or GSH and organic pollutants was found at the River Vltava. However, majority studies have not confirmed those which show higher GST activity related to organic pollutants (Vanderoost *et al*, 1994; Vigano *et al*., 1995; Petrivalsky *et al*., 1997; Havelkova *et al*., 2008a). This variable results could be the effect of fish species' differing sensitivity to the presence of contaminants in the aquatic environment (Hamed *et al*., 2003).

The correlation between liver biochemical markers and their inductors was statistically significant in some cases (the River Tichá Orlice, the River Elbe and its tributaries). It was confirmed that these biomarkers are very suitable and useful tool for monitoring of aquatic environment pollution in the rivers in the Czech Republic. Except this positive correlation, a decrease of biomarkers′ activity was determined at some highly polluted locations (e.g. on the River Vltava) – these results are in accordance with contemporary findings about protective mechanisms in aquatic organisms living in highly polluted environment who defend against exhaustion of enzymatic reserves stocks (Brammell *et al*., 2004). The different levels of enzymatic activity observed in some fish could be related to the typical CYP1A induction in fish, where small differences in the level of exposure to CYP1A inducers may give rise to great differences in enzymatic activity. In natural fish populations, the combined influence of biotic and abiotic factors are known to cause background variations in CYP 450 levels and activities (Goksoyr and Forlin, 1992).

It was proven that the use of the selected biochemical markers and chemical analyses has been a suitable tool to monitor the level of contamination of aquatic environment.
